# MicroRNA-128-3p Enhances the Chemosensitivity of Temozolomide in Glioblastoma by Targeting c-Met and EMT

**DOI:** 10.1038/s41598-020-65331-3

**Published:** 2020-06-11

**Authors:** Chengbin Zhao, Ruiming Guo, Fangxia Guan, Shanshan Ma, Mu Li, Junru Wu, Xianzhi Liu, Hongwei Li, Bo Yang

**Affiliations:** 1 Department of Neurosurgery, The First Affiliated Hospital of Zhengzhou University, Zhengzhou, 450052 Henan China; 2 School of Science, Zhengzhou University, Zhengzhou, 450001 Henan China

**Keywords:** CNS cancer, CNS cancer

## Abstract

Temozolomide is a first line anti-tumor drug used for the treatment of patients with Glioblastoma multiforme (GBM). However, the drug resistance to temozolomide limits its clinical application. Therefore, novel strategies to overcome chemoresistance are desperately needed for improved treatment of human GBM. Recent studies have demonstrated that miRNAs are closely related to resistance to cancer chemotherapy. This study aimed to further validate the biological role of miR-128-3p and to investigate whether miR-128-3p can enhance the chemosensitivity of glioblastoma to temozolomide (TMZ) and the underlying mechanisms. The effects of miR-128-3p and TMZ on the proliferation of glioblastoma cells were investigated by cell counting kit-8 (cck8). Transwell and intracerebral invasion assays were applied to determine the effects of the combination of miR-128-3p and TMZ on the invasion and migration of glioblastoma *in vitro* and *in vivo*. Flow cytometry was used to detect apoptosis in each group, and immunofluorescence was used to determine the expression levels of EMT-related proteins. RT-PCR and Western-blot were applied to detect EMT-transformed proteins (c-Met, PDGFRα, Notch1, and Slug) and EMT phenotype-associated proteins (Vim, CD44, and E-cadherin) at both mRNA and protein levels. Based on the microRNA.org database, we predicted the target genes of miR-128-3p. The target-relationship between miR-128-3p and c-Met and PDGFRα was verified by dual luciferase reporter gene. The tumor volume, weight and the expression levels of the proteins described above were measured in subcutaneously transplanted tumor model in nude mice. We found that the expression of miR-128-3p was down-regulated in glioblastoma tissue samples and cell lines. miR-128-3p suppressed the proliferation, migration, and invasion of GBM both *in vitro* and *in vivo*; miR-128-3p enhanced the therapeutic effect of TMZ via inhibition of proliferation, invasion and migration of glioblastoma cells and induction of apoptosis. Overexpression of miR-128-3p down-regulated the expression levels of EMT-transformed proteins (c-Met, PDGFRα, Notch1 and Slug) to enhance the effect of TMZ. In addition, we found that miR-128-3p targeted and bound c-Met. More importantly, the upregulation of c‐Met significantly prompted U87 and U251 cell proliferation. This effect could be abolished when c‐Met was silenced. The investigation in tumor bearing nude mice showed that miR-128-3p in combination with TMZ reduced tumor volume and the invasion extent, and increased the sensitivity of glioblastoma to TMZ. miR-128-3p is capable of enhancing the sensitivity of glioblastoma to TMZ through regulating c-Met/EMT.

## Introduction

Glioblastoma is the most common intracranial tumor in neurosurgery with poor prognosis and high mortality. TMZ chemotherapy can prolong the overall survival for patients with glioblastoma. However, due to intratumoral heterogeneity, there exist the primary drug-resistant glioblastoma stem cells (GSCs) and epithelial-mesenchymal transition (EMT) cells in glioblastoma, and drug-resistant subgroups after chemotherapy. Re-growth of tumor leads to tumor recurrence or metastasis^1^. If the subgroup is a homogenous epithelial subtype, then glioma cells are equally sensitive to the given treatment, and a precise and personalized plan can be developed.

MicroRNAs (miRNAs) are endogenous 18–25 nt non-coding RNA molecules that bind to the 3′-untranslated (3′UTR) seed region of their target gene and negatively regulate the expression of their target genes^2,3^. MiR-128-3p plays an important regulatory role in the development of many types of tumors^4^, including prostate cancer, lung cancer, MLL-AF4 acute lymphocytic leukemia, colorectal cancer, and breast cancer^5–9^. miR-128-3p is closely associated with clinical prognosis in patients with glioblastoma^10,11^, and it inhibits proliferation, migration, and invasion of glioma cells by directly targeting RhoE, BMI1, and E2F3^12,13^. It has been found that a variety of miRNAs can enhance the therapeutic effect of traditional drugs, but the underlying mechanism and whether miR-128-3p can also enhance the therapeutic effect of TMZ still remains unclear.

EMT occurs as a dynamic process that regulates the process of transforming “polarized epithelial cells” into “mesenchymal cells”. A wide variety of signaling pathways can influence this process^14,15^. EMT can be determined by loss of epithelial markers (e.g. E-cadherin) and up-regulation of mesenchymal markers (e.g. N-cadherin, fibronectin and vimentin)^14,16^. EMT can promote the migration and invasion of cancer cells, leading to drug resistance of tumor cells^17^. Thus, EMT inhibitors can be applied to prevent and reverse the EMT process through synergistically killing GSCs and EMT cells. Therefore, EMT inhibitors in combination of traditional drugs can be applied to enhance the effect of traditional chemotherapy. In fact, it has been reported that multiple miRNAs can inhibit EMT^18^. However, whether miR-128-3p can also inhibit EMT and enhance the therapeutic effect of TMZ also remains to be clarified.

c-Met is a receptor tyrosine kinase (RTK) protein encoded by the c-Met proto-oncogene and functions as a membrane receptor essential for embryonic development^19^. It was found that abnormal activation of c-Met in brain tumors induced cell proliferation, promoted tumor angiogenesis, inhibited cell death, induced tumor invasion, and promoted cancer stem cells to enhance glioma growth^20^. Overexpression of c-Met was also found to modulate chemosensitivity, leading to drug resistance in glioblastoma multiforme (GBM) cells and resulting in poor efficacy and the shortened survival time^21,22^. Inhibition of endogenous c-Met was reported to inhibit glioma growth and angiogenesis in the brain of nude mice and to promote apoptosis^23^. Thus, c-Met has become a promising therapeutic target^24^. Several inhibitors targeting c-Met have entered clinical trials^25–27^. However, whether miR-128-3p can regulate c-Met and alter TMZ sensitivity remains to be determined. Therefore, the purposes of this study were (1) to further validate the role of miR-128-3p in glioma; (2) to find out a new c-Met inhibitor; (3) to examine the relationship between miR-128-3p and c-Met and EMT; and (4) to elucidate whether miR-128-3p can increase the sensitivity of glioblastoma to TMZ and the underlying mechanism.

## Materials and Methods

### Cell lines

U87, T98, LN229, U251 and A172, were purchased from American Type Culture Collection (ATCC) (Manassas, VA, USA), HA1800 were purchased from Shanghai Honsun Biological Technology Co., Ltd (Shanghai, China).

### Materials

TMZ dissolved in DMSO, working concentration is 1μmol/L, was obtained from Sigma (Cat. T2577-25MG), DMSO was obtained from Sigma (Cat. D2650-5 × 5ML), Fetal bovine serum (FBS), DMEM medium, streptomycin, PBS and trypsin were obtained from GIBCO (Gaithersburg, MD, USA).

### Human glioblastoma tissue samples

Gliomablastoma and adjacent non-tumor tissues were obtained from the First Affiliated Hospital of Zhengzhou University (Zhengzhou, Henan, China, 2014-2018). According to the preoperative magnetic resonance film results and intraoperative observation, the tumor tissues and their matched adjacent normal tissues (n = 24) were excised during microsurgery. After being confirmed with immunohistochemical staining, all the samples were immediately frozen in liquid nitrogen and used for subsequent quantitative real-time polymerase chain reaction (RT-PCR) analysis. The study protocol was approved by the Ethics Committee of the First Affiliated Hospital of Zhengzhou University and was approved by all the patients participated in this research. Informed consent was obtained from all subjects or, if subjects are under 18, from a parent and/or legal guardian, and confirmed that all methods and experiments were performed in accordance with relevant guidelines and regulations

### Cell culture and transfection

Six human glioma cell lines, including U87, T98, LN229, U251, A172 and HA1800 were cultured in Dulbecco’s modified Eagle’s medium (DMEM) supplemented with 10% fetal bovine serum (FBS)(GIBCO, Gaithersburg, MD, USA) and 1% penicillin plus 1% streptomycin (GIBCO) were cultured in an incubator containing 5% CO_2_ at 37 °C. U87 and U251 cells were seeded into a 10 cm culture dish 1 day before infection. When the cell density reached 50% - 60% confluence, they were used for infection experiments. The LV-miR128-3p was used as the experimental group, and the LV-miR-NC was taken as the control group. The stably transfected cell lines were screened out for subsequent related experiments. Cells (3 × 10^5^ cells/well) were seeded in six-well plates and transfected with 100 nM/well of c-met siRNA or scramble siRNA performed with the X-tremeGENE siRNA transfection reagent, according to the manufacturer’s instructions (Roche, Basel, Switzerland). 48 hours after transfection, the cells were used for further experiments. miR-128-3p mimics (miR-128-3p), inhibitor (miR-128-3p-i) and negative control were synthesized by Genechem Company (Shanghai, China). c-met siRNA sequence: 5′-CCAAUGGAUCGAUCUGCCATT-3′; Scramble siRNA sequence: 5′-ACUACCGUUGUUAUAGGUGTT-3′. c-met over-expression plasmid vector was purchased from the Genechem Company.

### Wound-healing assay

Glioblastoma cells, U87 and U251 (3.0 × 10^5^) stably expressing miR-128-3p were seeded on a 6-well plate and cultured to form a monolayer. The cell layer was scraped with a sterile plastic tip. After being washed 3 times with phosphate buffered saline (PBS), the cells were then cultured in DMEM (Gibco) supplemented with 1% fetal bovine serum (Gibco). Images of the plates were captured under a microscope at 0 and 48 hours after scratching. Digimizer image analysis software was used to measure the distance between the two edges of the scratch.

### RNA extraction and RT-PCR

To determine the expression levels of genes encoding E-cadherin, CD44, Vimentin, c-Met, notch1, Slug and PDGFAa. Total RNA from tissue samples or cells were extracted by using TRIzol reagent (Invitrogen, Carlsbad, CA, USA) according to the manufacturer’s protocol. For miRNA reverse transcription, 1 μg of total RNA was reverse-transcribed into cDNA by using the All-in-One miRNA First-Strand cDNA Synthesis Kit (GeneCopoeia, Rockville, MD). For mRNA complementary DNA (cDNA) was generated with M-MLV reverse transcriptase (Invitrogen, Carlsbad, CA, USA) using RNA as a template. RT-PCR was performed in triplicate using the SYBR Green Master Mix Kit (Applied Biosystems, Foster City, CA, USA). GAPDH and U6 were used as internal controls for mRNA and miRNA RT-PCR, respectively. The 2^-ΔΔCt^ method was used to analyze the relative levels of gene expression. Each specimen was repeated biologically three times. The primer sequences used in this study are as follows:

Notch1, F1: GGACCAGATTGGGGAGTT, F2:CACACTCGTCCACATCGT; E-cadherin, F1: ATTCTGATTCTGCTGCTCTTG, F2: AGTCCTGGTCCTCTTCTCC; Vimentin, F1: AATGACCGCTTCGCCAAC, F2: CCGCATCTCCTCCTCGTAG; Slug, F1: AGATGCATATTCGGACCCAC, F2: CCTCATGTTTGTGCAGGAGA; CD44, F1: TGAATATAACCTGCCGCTTTG, F2: GCTTTCTCCATCTGGGCCAT; PDGFRα, F1: ATCAATCAGCCCAGATGGAC, F2: TTCACGGGCAGAAAGGTACT; c-Met, F1: AGTGGGAATTCTAGACACATTTCA, F2: CATTCAAGAATACTGTTTGACACACTT; GAPDH, F1: GATTCCACCCATGGCAAATTCC, F2: CACGTTGGCAGTGGGGAC; miR-128-3p: F1: GGTCACAGTGAACCGGTC, F2: GTGCAGGGTCCGAGGT; U6, F1: CTCGCTTCGGCAGCACATATACT, F2: ACGCTTCACGAATTTGCGTGTC;

### Western blot

The protein expression levels of E-cadherin, CD44, Vimentin, c-Met, notch1, Slug and PDGFAa were measured by Western-blot. The cultured cells were firstly washed twice with pre-chilled PBS buffer and then lysed with RIPA buffer (Sigma-Aldrich, St. Louis, MO, USA) containing a set of protease inhibitors and total protein was extracted. The protein concentration was determined using a BCA protein assay kit (Pierce Chemicals Co, Dallas, TX, USA), and then separated by electrophoresis on a 10% sodium dodecyl sulfate polyacrylamide gel electrophoresis (SDS-PAGE) gel. The gel was transferred to a polyvinylidene difluoride (PVDF) membrane, which was then blocked with TBST containing 5% skim milk powder. The corresponding primary antibody for each of the proteins mentioned above was added and incubated at 4 °C overnight at dilutions as follows: E-cadherin (1:1000, CST, 14472), CD44 (1:1000, CST, 5640), Vimentin (1:1000, CST, 5741), c-Met (1:1000, CST, 3127), notch1 (1:1000, CST, 3608), Slug (1:1000, CST, 9958), PDGFAa (1:500, CST, 3164), and GAPDH (1:500, Abcam, 16039), respectively. The membrane was then incubated with horseradish peroxidase (1:2000, Abcam, 205718)-labeled secondary antibody for 2 hours at room temperature. The fluorescence intensity was then measured by a commercial Immobilon Western HRP Substrate (WBKLS0500, Millipore, USA) under dark conditions. GAPDH was used as an internal control. Each specimen was repeated three times.

### CCK-8 determination

After being given different treatments, viability rates of cells were determined by CCK-8 kit (Beyotime Biotechnology, Haimen, Jiangsu, China). The cells in each group were digested with trypsin and a single cell suspension was prepared, which was then seeded into 96-well plates (Corning, New York, NY, USA) with a density of 3000 cells/well. Each group of cells was set with 4 replicate wells and cultured in an incubator. The culture medium was replaced with a serum-free medium containing 10 μl of CCK-8 solution (Beyotime Biotechnology, Haimen, Jiangsu, China) for 1, 2, 4, and 5 days, and incubated for 2 h. The absorbance at a wavelength of 570 nm (OD_570_) was recorded. Each sample was repeated three times.

### Detection of apoptosis

Cells were seeded in six‐well plates (3 × 10^5^ cells/well) and then were treated with aptamer for 48 hours. After washing with PBS. The apoptosis rates of cells from different treatment groups were analyzed by Annexin V-FITC/PI Apoptosis Detection Kit (Beyotime Biotechnology, Haimen, Jiangsu, China). The cells were digested with trypsin without EDTA, collected by centrifugation at 1000 g for 5 min, adjusted to a cell concentration of 1 × 10^6^ cells/ml, and washed twice with PBS. 100 μl of 1× binding buffer, 5 μl of Annexin V-FITC and 5 μl of PI were added, respectively. After being mixed well, the reaction was allowed to stand at room temperature for 15 min in the dark, 500 μl of 1× binding buffer was then added. The apoptosis rate was detected in the dark for 1 h. All samples were immediately measured on FACSort flow cytometry (BD, USA). Both early and late apoptotic cells were recorded as apoptotic cells, and the results were expressed as the percentage of total cells. All the assays were repeated in triplicate.

### Cell invasion and migration assay

U251 and U87 cells from different treatment groups were harvested and collected by centrifugation at 1000 g for 5 min and then adjusted to a concentration of 1×10^6^ cells/ml; 600 μl of medium containing 10% fetal bovine serum was added to a 24-well culture plate (Corning, New York, NY, USA). 300 μl of the above prepared cell suspension was added to the chamber, and cultured in a CO_2_ incubator for 12 h; a 24-well culture plate containing a Transwell chamber was taken out, and the remaining medium in the chamber was discarded, and 1% crystal violet was added. The cells were stained at room temperature for 30 min and washed with PBS three times to wash off the excess dye. The unmigrated cells, which were in the upper chamber, were gently wiped with a cotton wool ball. The cells were observed and photographed under an inverted microscope (Olympus, Tokyo, Japan). For the invasion assay, the Transwell chamber was pre-coated with Matrigel (Becton Dickinson) before the cell suspension was seeded, and the remaining cells were migrated. The number of migrated cells was counted with Image J software. All the assays were repeated in triplicate.

### Immunofluorescence analysis

The cells of the different treatment groups were harvested and collected by centrifugation at 1000 g for 5 min, fixed with 4% paraformaldehyde for 15 minutes, permeabilized with 0.05% Triton X-100 for 10 minutes, blocked with 5% goat serum for 1 h, and incubated with the corresponding primary antibody, mouse anti-Vimentin (1:100, CST, 5741), mouse anti-E-cadherin (1:50, CST, 14472), mouse anti-α-Tubilin (1:2000, CST, 3873), mouse anti-F-actin (1:100, abcam, ab205), for 1 hour, followed by incubation with the corresponding secondary antibody, cy3-labeled Goat anti-mouse IgG antibody(1:200, abcam, ab97035), FITC-labeled Goat anti-mouse IgG antibody (1:100, abcam, ab6785), for 1 hour in the dark. The nuclei were stained with 4, 6-diamino-2-phenylindole (DAPI) (Beyotime Biotechnology, Haimen, Jiangsu, China). Images were acquired, observed and collected under a fluorescence microscope. Each sample was repeated three times.

### Dual luciferase assay

The dual luciferase reporter gene was applied to detect the targeted regulation of miR-128-3p on c-Met and PDGFRα. 293 T cells were seeded in 6-well plates and used for transfection when the cell density reached 50% to 60% confluence. The experiment was designed with 8 groups, each group consisted of 3 duplicate wells: (1) miR-NC + c-Met-WT; (2) miR-128-3p + c-Met-WT; (3) miR-NC + c- Met-mut; (4) miR-128-3p + c-Met-mut; (5) miR-NC + PDGFRα-WT; (6) miR-128-3p+ PDGFRα-WT; (7) miR-NC + PDGFRα- Mut; and (8) miR-128-3p + PDGFRα-mut. After the cells were seeded and incubated for 12 hours, the medium was changed. 48 hours later, Luciferase activity was measured using the Dual Luciferase Reporter Assay System as described by the manufacturer (Promega, Madison, WI, USA). Results shown are from three biological replicates, each performed with three technical replicates

### Animal experiments

Under the condition of no specific pathogen, 6-8 weeks old SCID mice were selected for the experiment. For brain invasion test, U251 cells highly expressing miR-128a were stained with 1,1′-dioctadecyl-3,3,3′,3′-tetramethylindocarbocyanine perchlorate (a cell membrane green fluorescent probe, DIL) at 10 μM. LV-miR-128-3p group) and control U251 cells were stained with 3,3′-dioctadecyloxacarbocyanine perchlorate (a cell membrane red fluorescent probe, DIO at 10 μM, LV-miR-NC group), respectively, and then mixed in 1:1 ratio. 2 × 10^5^ ells (100 μl) were injected into the brain of SCID mice stereotactically (The anterior fontanelle was used as the reference point, 2 mm on the right, 1 mm in the back, and 2.5 mm vertically), and TMZ (40 mg/kg/d) was injected once every three days for a total of 3 doses. After 7 days, the distance of migration of the two types of cells in the brain was observed. In the subcutaneous tumor-bearing experiment, 5 × 10^6^ cells were inoculated subcutaneously into the right forelimb of SCID mice. After the tumor had grown in SCID mice for 7 days, the tumor-bearing SCID mice were randomly divided into 4 groups with 6 mice in each group: (1) injection of normal saline + LV-miR-NC (control group); (2) injection of normal saline + LV-miR-128-3p (LV-miR-128-3p); (3) injection of TMZ + LV-miR-NC (LV- miR- NC + TMZ); and (4) Injection of TMZ + LV-miR-128-3p (LV-miR-128-3p + TMZ group). TMZ (40 mg/kg/d) was injected once every three days for a total of six total doses. The size of the tumor was measured every 4 days using a vernier caliper, and the survival time of the nude mice was observed. The nude mice were sacrificed 40 days after transplantation, and the tumor samples were taken out and used for subsequent measurement of tumor size and parallel phenotypic differentiation assay.

The animal experiments were approved and performed in accordance with the Animal Care and Use Committee of the First Affiliated Hospital of Zhengzhou University.

### Statistical analysis

In this study, all the measurements were expressed as mean ± standard error (SE). An independent sample t-test was used to analyze the difference in mean values between the two groups. One-way ANOVA was used to compare the differences in mean values among three or more groups. Two-tailed *P* < 0.05 was considered statistically significant. All the statistical analyses were performed using SPSS 18.0 software.

### Ethics approval and consent to participate

The study protocol was approved by the Ethics Committee of the First Affiliated Hospital of Zhengzhou University and was approved by all the patients participated in this research.

## Results

### miR-128-3p was down-regulated in human GBM tissues and cell lines

**Figure 1:**
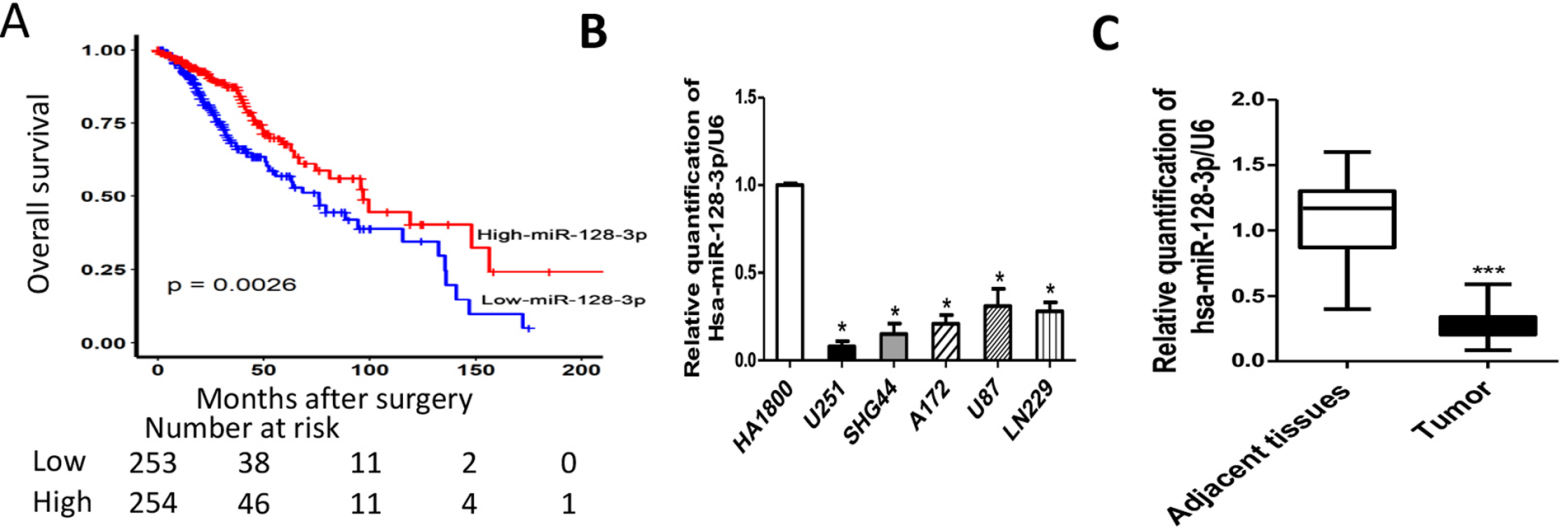
Expression of miR-128-3p in glioma cell lines and tissues and *in vitro* and *in vivo* functions. (**A**) TCGA analysis of the expression of miR-128-3p and prognosis (n = 620, p = 0.0026); (**B**) RT-PCR detection of expression of Hsa-miR-128-3p gene in glioma cell lines U251, SHG44, A172, U87, LN229 and HA1800. U6 were taken as an internal reference gene, compared with HA1800 (**C**) The relative expression level of hsa-miR-128-3p in glioma tissues (tumor) and their matched adjacent normal tissues was examined by RT-PCR. The date were presented as fold change. normalized by U6, n = 24. Compared with the control group, * means *P* < 0.05, *** means *P* < 0.001.

To investigate the expression level of miR-128-3p in GBM, we analyzed the data (n = 620) from TCGA online data set (http://cancergenome.nih.gov/). Univariate and multivariate analyses showed that miR-128-3p was significantly associated with glioma prognosis (Supplementary Table S1). We used Kaplan–Meier survival curves to analyze the overall survival rates of patients with GBM and found that the overall survival rates of patients with low levels of miR-128-3p were significantly worse than those of GBM patients with relatively high levels of miR-128-3p (*P* = 0.0026) (Fig. 1A). To further examine the expression level of miR-128-3p in GBM, we compared miR-128-3p levels in five GBM cell lines, namely U251, SHG44, A172, U87 and LN229, and an immortalized human normal glioma cell line, HA1800. The miR-128-3p expression levels were significantly lower in all five GBM cell lines as compared with that of the normal cell line (*P* < 0.01) (Fig. 1B). The expression level of miR-128-3p was also strongly down-regulated in human GBM tissues as compared with their normal counterparts (Fig. 1C). All of these data suggested that miR-128-3p was down-regulated in human GBM tissues and GBM cell lines.

### miR-128-3p suppressed the proliferation, migration, and invasion of GBM both *in vitro* and *in vivo*

**Figure 2:**
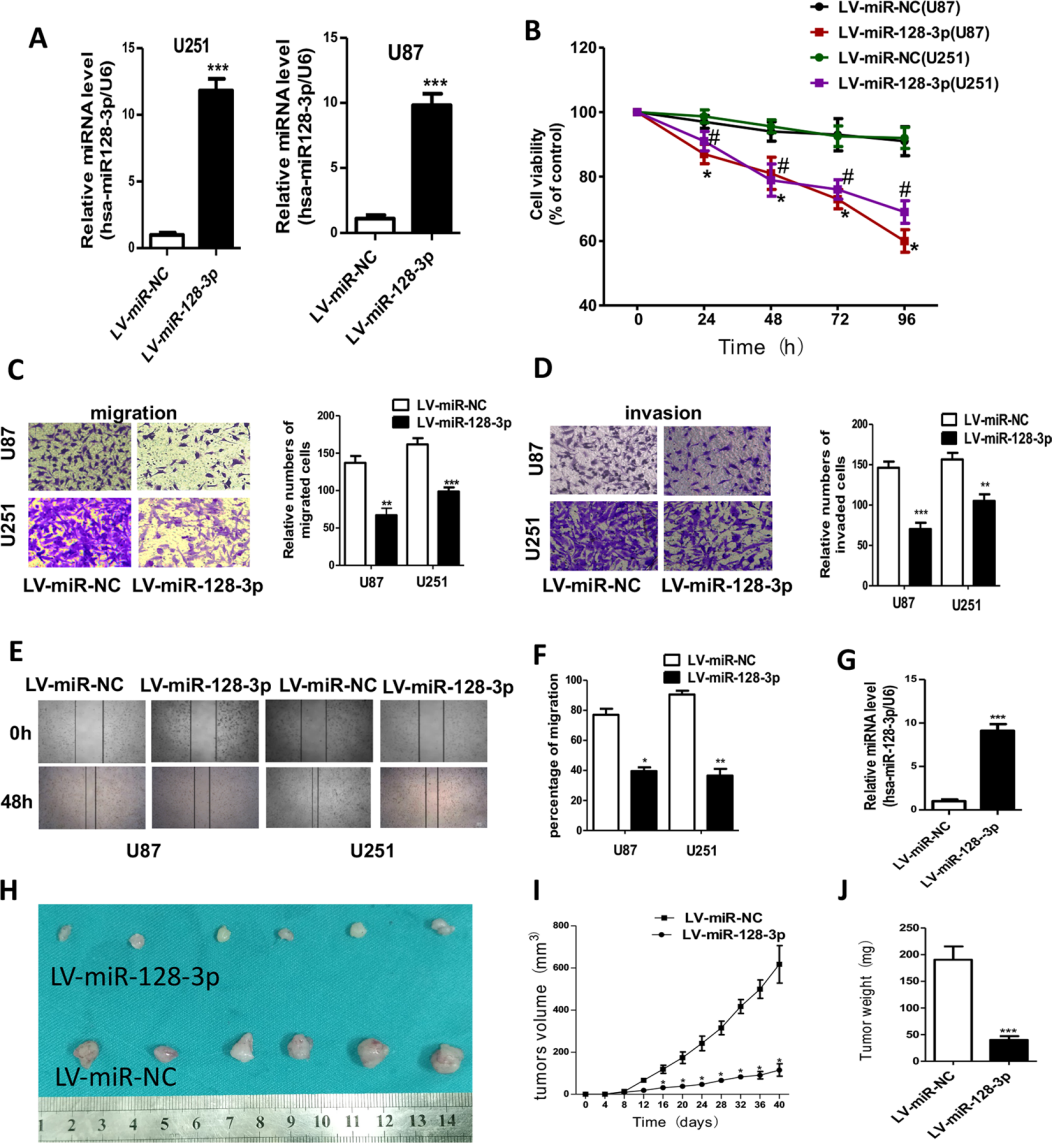
miR-128-3p suppressed the proliferation, migration and invasion of GBM *in vitro* and *in vivo*. (**A**) RT-PCR was used to verify the expression of miR-128-3p in stably transfected U87 and U251 cells; (**B**) cck-8 detects cell viability; (**C**,**D**) Transwell detects invasion, migration and statistical analysis, (**E**,**F**) wound healing experiments and statistical analysis, (**G**) RT-PCR was used to verify the expression of miR-128-3p gene, U6 was taken as an internal reference gene; (**H**) tumor-bearing results in nude mice, (**I**) tumor growth curve analysis; and (**J**) statistical analysis of tumor weight. Each sample was repeated in triplicate. Data are expressed as mean ± standard error. Compared with the LV-miR-NC group, * means *P* < 0.05, ** means *P* < 0.01, and *** means *P* < 0.001, ^**#**^ means P < 0.05.

In order to determine the biological functions of miR-128-3p, we established an U87 cell line and an U251 cell line stably expressing miR-128-3p, respectively, and verified their status by RT-PCR (Fig. 2A), we performed CCK8 assay to explore the effect of miR-128-3p on the proliferation of glioblastoma cells. The results showed that up-regulation of miR-128-3p significantly reduced the proliferation rates of both U251 and U87 cells (Fig. 2B). Overexpression of miR-128-3p significantly reduced the invasion and migration of both U251 and U87 cells (Fig. 2C,D). Wound healing experiments showed that overexpression of miR-128-3p inhibited wound healing areas of U251 and U87 cells (Fig. 2E,F). After RT-PCR verification of the subcutaneous tumor-bearing experiments in nude mice (Fig. 2G), the results (Fig. 2H–J) showed that the tumor volume and weight of miR-128-3p group were significantly reduced compared with those of the control group.

### miR-128-3p suppressed GBM cell proliferation, migration and invasion to enhance the chemosensitivity of temozolomide *in vitro*

**Figure 3:**
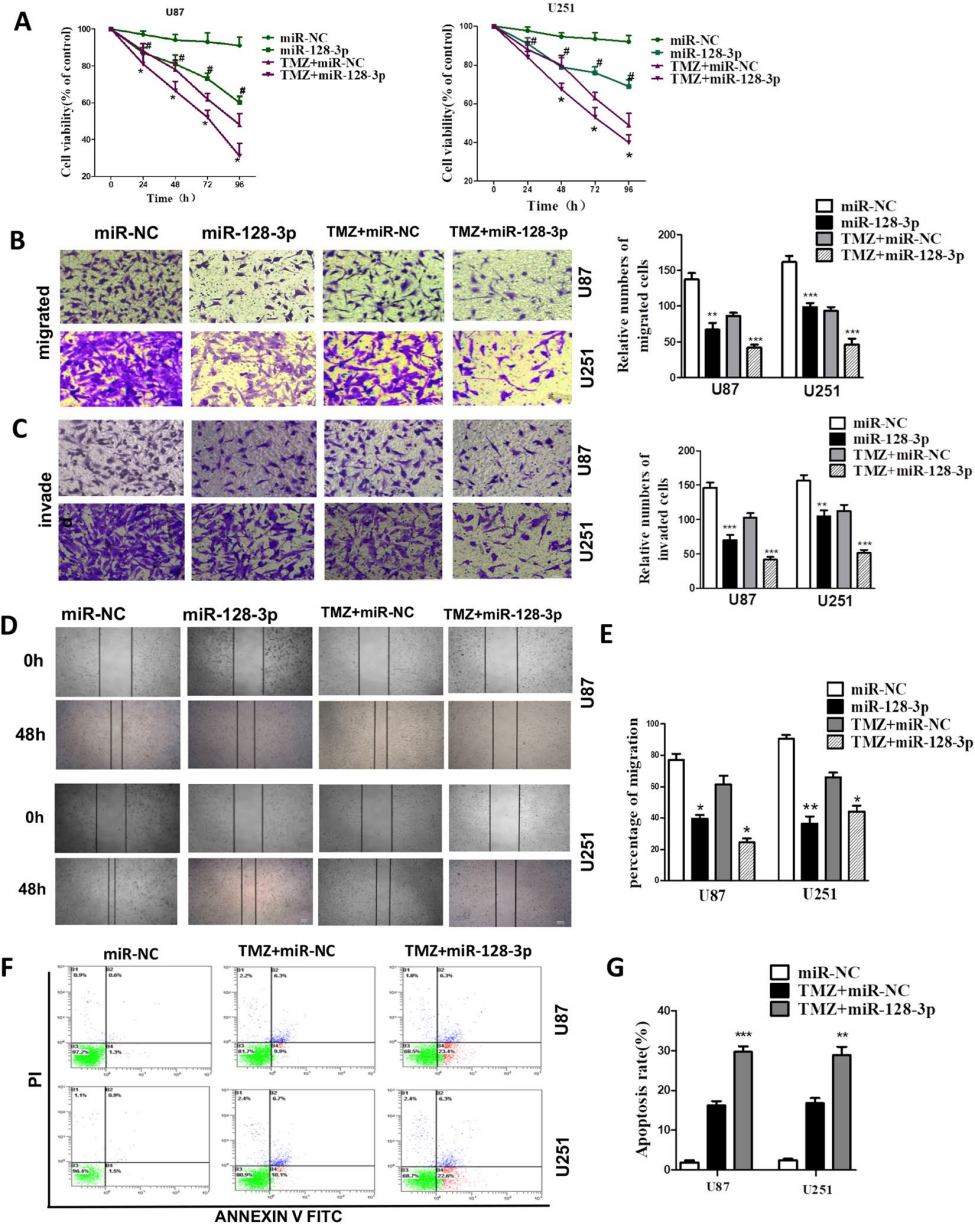
miR-128-3p increases the effect of TMZ *in vitro*. TMZ: Temozolomide (**A**) cck-8 for cell viability, (**B**,**C**) Transwell for cell migration, invasion, and statistical analysis (**D**,**E**) wound healing assay for migration and statistical analysis, (**F**,**G**) Detection of apoptosis and statistical analysis with flow cytometry. Each sample was repeated in triplicate. Data are expressed as mean ± standard error. Compared with the LV-miR-NC group, * means *P* < 0.05, ** means *P* < 0.01, and *** means *P* < 0.001.

To investigate whether miR-128-3p is capable of enhancing the efficacy and mechanism underlying TMZ chemotherapy, we firstly performed the *in vitro* experiments. The cck8 assays showed that in glioma U87 and U251 cells, the cell viability of miR-128-3p + TMZ group was significantly reduced as compared with that of miR-NC + TMZ group (Fig. 3A), indicating that miR-128-3p in combination with TMZ is more effective than TMZ alone. Through the scratch test and Transwell experiment, the wound healing area of the miR-128-3p in combination with TMZ group was smaller than that of TMZ group (Fig. 3B,C), and the number of transmembrane cells was also significantly decreased (Fig. 3D,E), indicating that miR-128-3p can reduce the migration and invasion ability of GBM cell lines U251 and U87. Detection of apoptosis with flow cytometry showed that the total apoptosis rate in miR-128-3p + TMZ group (28%) was higher than that of miR-NC + TMZ group (16%) (Fig. 3F,G), confirming that miR-128-3p can enhance the effect of TMZ by increasing apoptosis in glioblastoma cells.

### MiR-128-3p enhanced the chemosensitivity of glioblastoma to temozolomide *in vivo* by suppressing GBM cell proliferation and invasion

**Figure 4:**
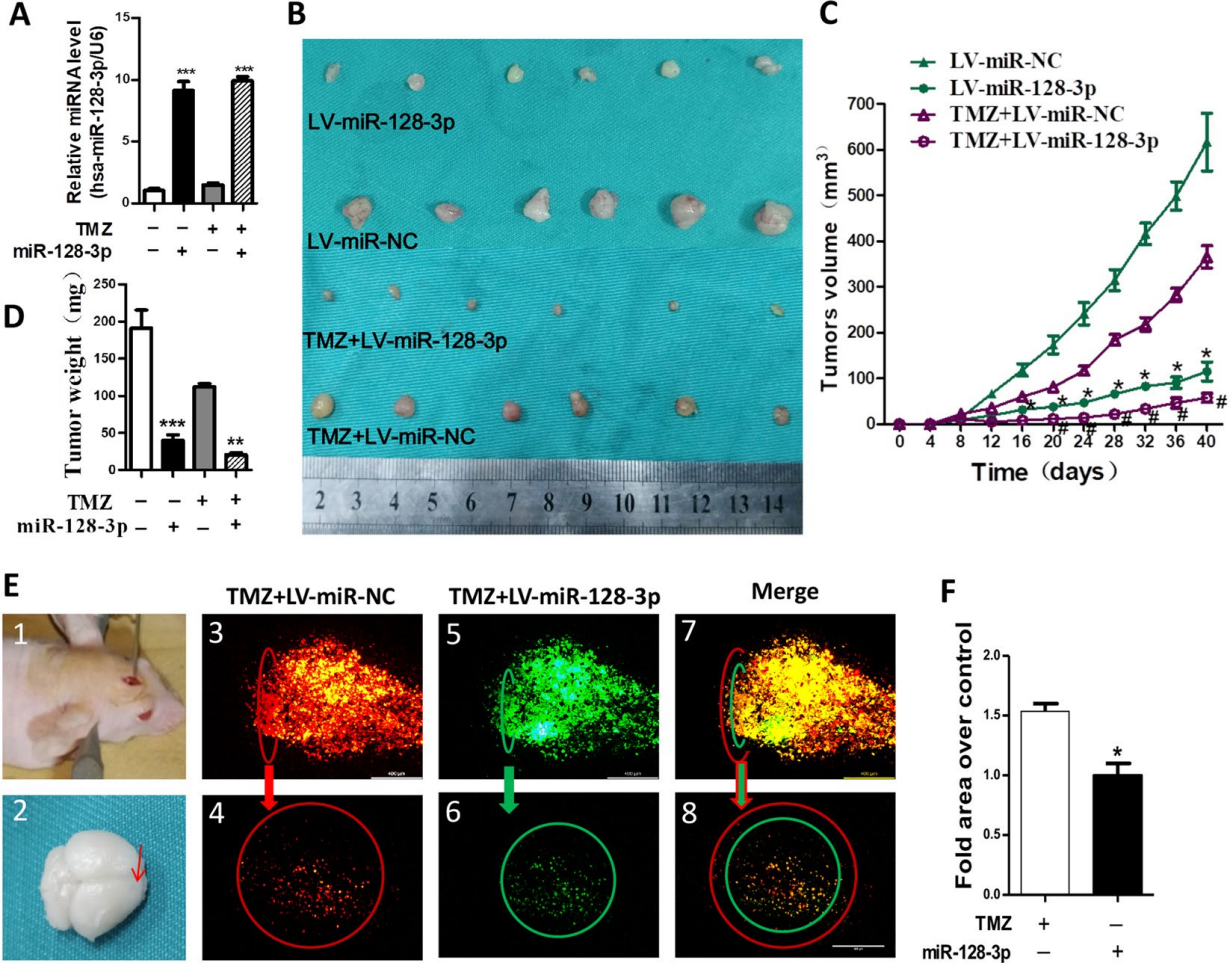
miR-128-3p increases the effect of TMZ *in vivo*. (**A**) RT-PCR to verify miR-128-3p gene expression, U6 as an internal reference gene; (**B**) Photographs of the dissected tumors. (**C**) tumor growth curve analysis; (**D**) tumor weight statistical analysis; (**E**) U251 brain invasion test, (1) Stereotactic injection of mixed U251 cells (LV-miR-NC group (DIL-red) + LV-miR-128-3p group (DIO-green)) (2) intracerebral injection sites; (3) and (5) same sagittal plane fluorescence imaging, (4) and (6) same cross-sectional fluorescence imaging, The red circle represents the intracerebral invasion range of the LV-miR-NC group, and the green circle represents the intracerebral invasion range of the LV-miR-128-3p group, (7) and (8) Sagittal and cross-sectional merge imaging; (**F**) statistical analysis of intracerebral invasion experiments, each sample was repeated three times. Data are expressed as mean ± standard error. Compared with the TMZ + LV-miR-NC group, * means P < 0.05, ** means P < 0.01, and *** means P < 0.001.

In order to further verify whether miR-128-3p can play the same role *in vivo*, U251 cells stably expressing miR-128-3p were subcutaneously implanted in nude mice. RT-PCR analysis confirmed that the expression levels of miR-128-3p in TMZ + LV-miR-128-3p group were higher than that in TMZ + LV-miR-NC group in tumor tissues (Fig. 4A). The results showed that the tumor volumes of LV-miR-128-3p + TMZ were significantly reduced as compared with that of LV-miR-NC + TMZ group (Fig. 4B–D). To verify the invasive effect in the brain, a brain invasive experiment in nude mice was performed. The results showed that under the same field of view, invaded area of U251 cells in the LV-miR-128-3p + TMZ group was significantly reduced as compared to that of LV-miR-NC + TMZ group (Fig. 4E,F).

### miR-128-3p inhibited expression of proteins involved in EMT and multiple emt pathway

**Figure 5:**
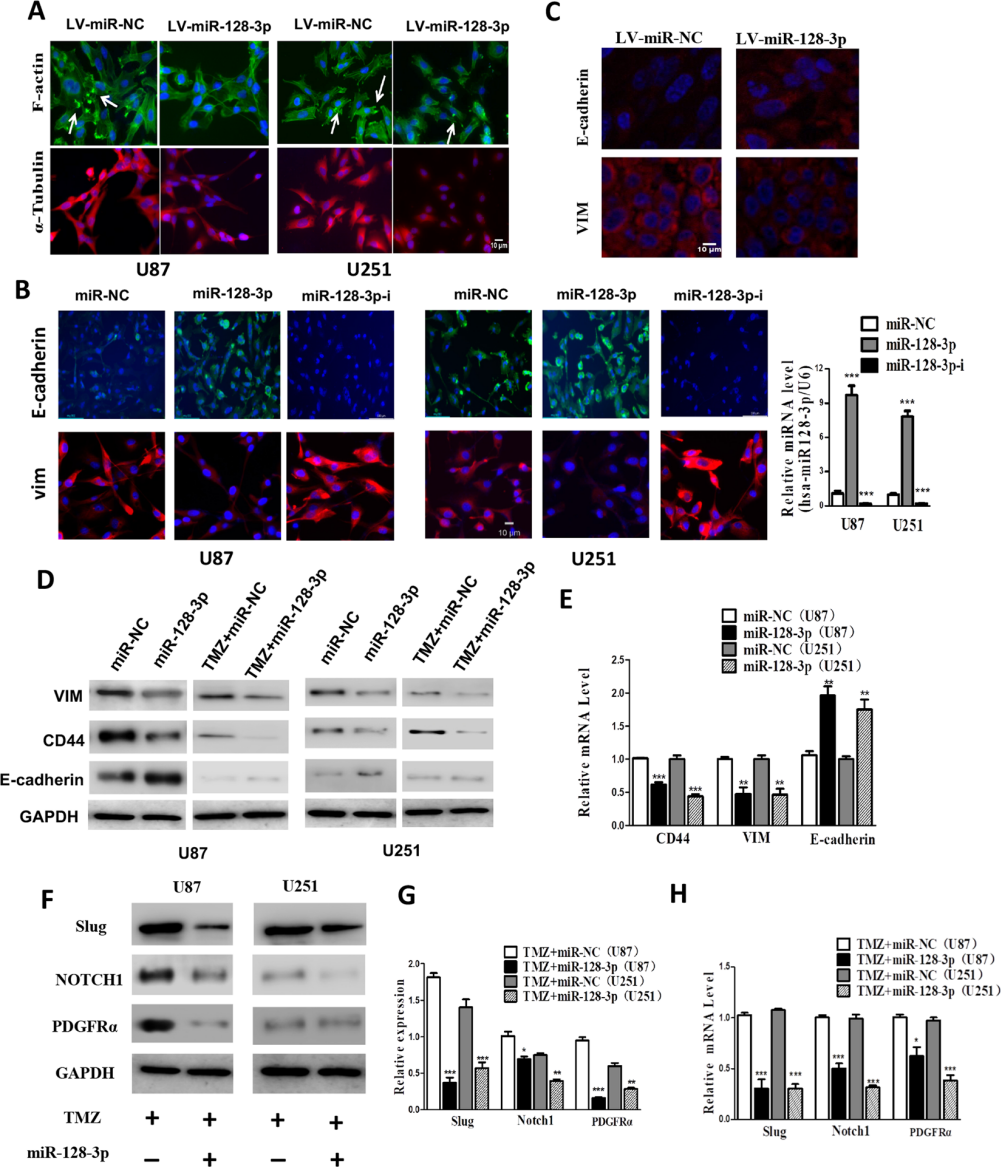
miR-128-3p increases TMZ chemosensitivity by inhibiting EMT and related pathways. (**A**) immunofluorescence analysis of α-Tubulin and F-actin expression; (**B**) immunofluorescence analysis of EMT-related protein VIM and E-cadherin expression; (**C**) immunofluorescence detection of subcutaneous tumor-bearing model EMT-related protein in nude mice VIM and E-cadherin expression; (**D**) Western-blot detection of EMT-related protein VIM, CD44, E-cadherin expression, GAPDH as an internal reference; (**E**) RT-PCR detection of EMT-related genes VIM, CD44, E-cadherin expression; (**F**) Western-blot detection of the expression of PDGFRα, Notch1 and Slug protein, GAPDH was taken as the internal reference, (**G**) Western-blot statistical analysis; (H) RT-PCR detection of the mRNA expression levels of PDGFRα, Notch1 and Slug genes. Each sample was repeated in triplicate. The data is expressed as mean ± standard error. Compared with the miR-NC group, * means P < 0.05, ** means P < 0.01, and *** means P < 0.001.

In order to study the mechanism by which miR-128-3p enhances the sensitivity of glioblastoma cells to TMZ, through immunofluorescence, we found that miR-128-3p reduced the adhesion of pseudopods and inhibited the cell movement of tubulin (Fig. 5A). These morphological changes can reduce the invasive ability of tumor cells. Immunofluorescence also revealed that miR-128-3p up-regulated the expression levels of epithelial marker E-cadherin in GBM cell lines U251 and U87 and down-regulated the expression of mesenchymal marker VIM, preventing EMT cell formation (Fig. 5B), thereby enhancing the biological effect of TMZ. The killing effect of the drug on GBM was the same in the immunofluorescence test of the tumor-bearing specimens of nude mice (Fig. 5C). Furthermore, Western-blot and RT-PCR analysis showed the same trend in GBM cell lines U251 and U87 (Fig. 5D,E). It was also observed that miR-128-3p in combination with TMZ down-regulated the expression levels of PDGFRα, Notch1 and Slug in GBM cell lines U251 and U87 (Fig. 5F,G). These key proteins can promote EMT via different pathways. RT-PCR analysis also presented the same results (Fig. 5H).

### miR-128-3p inhibits glioma cell viability by directly targeting c-Met

**Figure 6:**
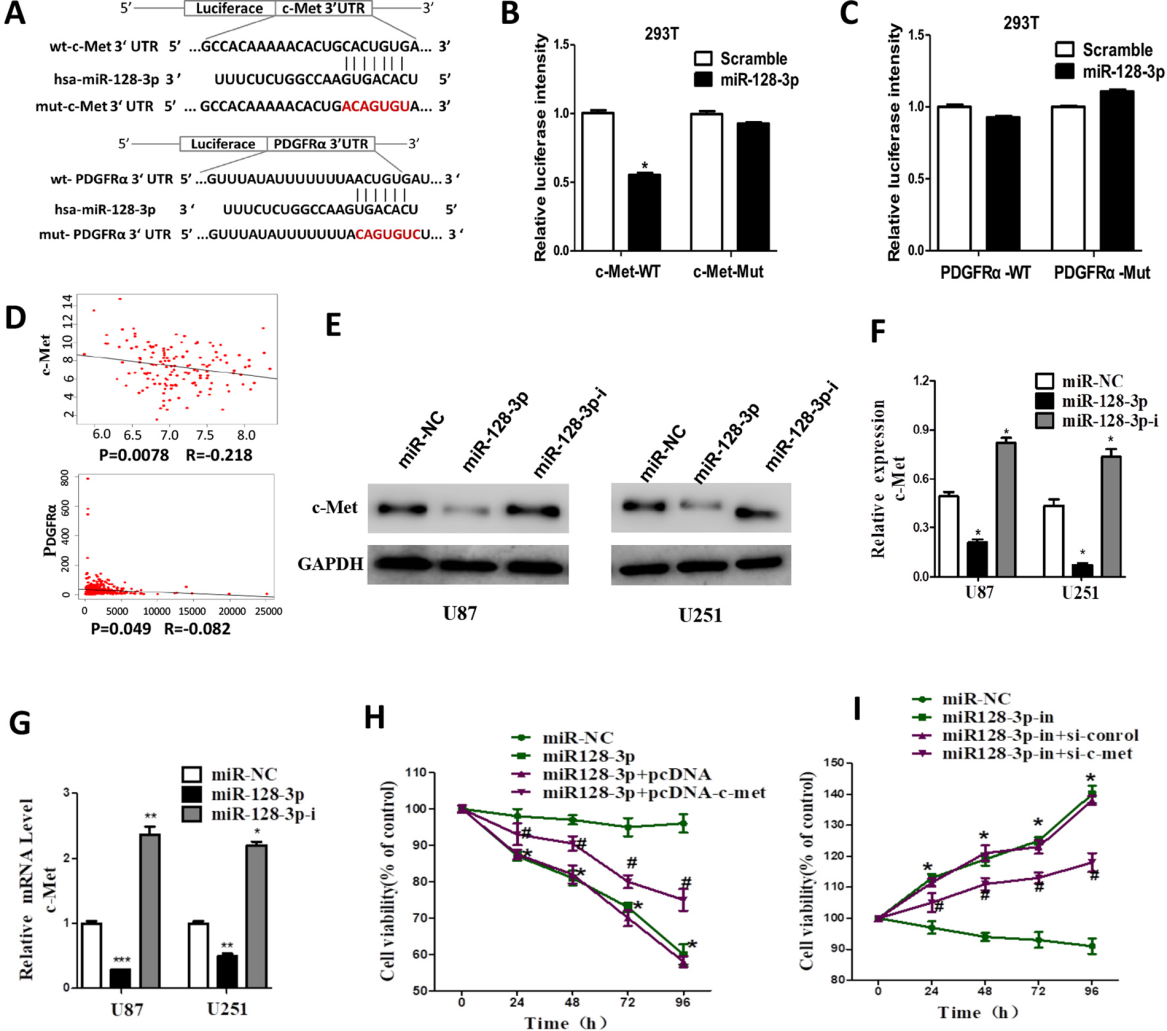
miR-128-3p directly targets c-Met. (**A**) MET and PDGFRα 3′UTR and Has-miR-128-3p targeted binding site; (**B**) c-Met dual luciferase statistical analysis; (**C**) Statistical analysis of PDGFRα dual luciferase; (**D**) Analysis of the relationship between miR-128-3p and c-Met and PDGFRα expression in TCGA database; (**E**) Western-blot detection of the expression of c-Met protein; (**F**) Western-blot statistical analysis; (**G**) RT-PCR was used to detect the expression level of c-Met mRNA (**H**,**I**) cck-8 for cell viability. Data are expressed as mean ± standard error. Compared with the miR-NC group, * means P < 0.05, ** means P < 0.01, and *** means P < 0.001.

TargetScan (http://www.targetscan.org/) was used to investigate the downstream target of miR-128-3p. We found that the c-Met and PDGFRα 3′ untranslated region (UTR) exhibited potential binding to miR-128-3p site (Fig. 6A), on which wild-type and mutant c-Met and PDGFRα eukaryotic expression vectors were constructed. The dual-luciferase reporter gene assay showed a significant decrease in the fluorescence activity of 293 T cells co-transfected with miR-128-3p + c-Met-WT (*P* < 0.01) (Fig. 6B). However, the fluorescence activity of 293 T cells co-transfected with miR-128-3p+PDGFRα was not significantly different (less than 20%) (Fig. 6C). These data indicated that the direct interaction between miR-128-3p and c-Met 3′UTR resulted in the decreased expression of c-Met mRNA. A survey of the TCGA dataset revealed a significantly negative correlation between miR-128-3p and c-Met mRNA expression (R = −0.218, *P* = 0.0078). PDGFRα also showed the same result (Fig. 6D). Western-blot analysis showed that overexpression of miR-128-3p reduced c-Met expression, while downregulation of miR-128-3p leads to increased expression of c-met (Fig. 6E,F). RT-PCR also confirmed the result (Fig. 6G). Overexpression of miR-128-3p, cell viability of glioma cell U87 was significantly reduced. However, overexpression of miR-128-3p simultaneously increased the expression of c-met, the cell viability effect of U87 was reversed. Changes in cell viability caused by down-regulation of miR-128-3p expression can also be reversed by silencing c-met. Decreasing the expression of c-met can increase the sensitivity of GBM cells to drugs and increase the effect of drugs, while down-regulation of c-Met can inhibit the proliferation of glioma cells.

## Discussion

The dysregulated expression of miRNAs are closely related to the malignant biological behaviors of tissues and cells^28,29^. The miR-128 is a highly expressed miRNA in the normal brain nervous system^30^. There is increasing evidence indicating that miR-128-3p plays an important role in the development of various types of cancers and diseases^31^ and with colloid clinical prognosis in patients with blastoma^10,11^. In the present study, through the *in vitro* and *in vivo* experiments, we further verified the biological role of miR-128-3p in glioblastoma, further confirming its capability of inhibiting tumor proliferation, invasion and migration. In the present study, we studied the relationship between miR-128-3p and EMT and the mechanism of enhancing the therapeutic effect of TMZ. Immunofluorescence assay revealed that miR-128-3p up-regulated the expression of epithelial marker E-cadherin and down-regulated the expression of mesenchymal marker VIM, preventing EMT formation. Through the combination experiments, we found that miR-128-3p in combination with TMZ significantly reduced the proliferation, invasion and migration of glioblastoma cells as compared with TMZ alone, confirming that miR-128-3p can enhance the inhibitory effects of TMZ in cell proliferation, invasion and migration by inhibiting EMT.

C-Met has been known to be highly expressed in a large number of tumors and has been used clinically as a standard therapy for patients with NSCLC^32^. The c-Met plays an important role in tumor progression and treatment^20,33^, regulates glioma proliferation and cell cycle^34^, regulates cancer stem cells^23,35^, and has recently become a functional marker of glioblastoma stem cell^23^. Targeting c-Met receptors for the treatment of thyroid cancer has entered clinical trials, with nearly 60% of patients receiving treatment having the reduced tumor mass^26^. The c-Met can also modulate chemosensitivity. Its overexpression led to drug resistance in GBM cells, resulting in poor efficacy and shortened survival time^22^. Overexpression of c-Met is related to the shortened survival time and the poor response of glioblastoma cells to therapy agents while down-regulation of c-Met can inhibit the proliferation, invasion and metastasis of glioma cells^22^. In addition, c-Met activates multiple downstream signaling pathways to induce EMT by reducing cell adhesion and increasing cell motility^33^, further enhancing tumor cell invasion.

Treatment of glioblastoma by targeting c-Met has also been used in phase II clinical trial studies, and the study found that all the patients receiving c-Met inhibitors had a total disease control rate approaching 50%^25,32^, which means that targeting the c-Met receptor is an effective strategy to increase the therapeutic effect on glioma. In this experiment, we studied the relationship between miR-128-3p and c-Met by bioinformatics and dual luciferase experiments, which have confirmed that miR-128-3p is an important regulator of the c-Met signal transduction pathway. In the present study, we found that miR-128-3p could down-regulate the expression of PDGFRα, Notch1 and Slug while the dual luciferase assay found that miR-128-3p did not directly bind to PDGFRα, and thus, it may confer the effect in an indirect way. This possibility needs to go further studied. Our experiments also found that miR-128-3p down-regulated the expression of Notch1 and Slug. Notch1 directly activates the Notch pathway^36–38^, while the transcription factor Slug directly inhibits EMT^39^. However, miR-128-3p binds to key genes involved in the above mentioned EMT pathway, down-regulates the expression of related proteins, inhibits the process of EMT, thereby reducing the production of “stem-like” cells and EMT cells and inhibiting cell proliferation, invasion and migration to exert its effects.

In summary, the main finding of this study was that miR-128-3p increased the sensitivity of glioblastoma to TMZ and thus, increased the therapeutic effect of TMZ. Overexpression of miR-128-3p increased the sensitivity of glioblastoma to TMZ by targeted inhibition of c-Met expression and inhibition of epithelial-mesenchymal transition (EMT). This study has provided new insights into the role of miR-128-3p in enhancing the chemosensitivity of glioblastoma and its underlying mechanisms. Thus, miR-128-3p can be a potential target for the development of new therapeutic strategies overcoming drug resistance. Development of a drug targeting miR-128-3p combined with TMZ chemotherapy can improve the effect of glioma and increase the prognosis of patients.

## Supplementary information

supplementary Table S1.

## Data Availability

All data are fully available without restriction. To further request the data, please contact professor Hongwei Li (fcclihw1@zzu.edu.cn)
