# Supplementary material for: MicroRNA-128-3p Enhances the Chemosensitivity of Temozolomide in Glioblastoma by Targeting c-Met and EMT
Source: Sci Rep. 2020 Jun 11;10:9471. doi: 10.1038/s41598-020-65331-3 (PMC7289811; doi:10.1038/s41598-020-65331-3)
Supplement: Supplementary file 1 — supplementary Table S1. [file 41598_2020_65331_MOESM1_ESM.docx]

MicroRNA-128-3p Enhances the Chemosensitivity of Temozolomide in Glioblastoma by Targeting c-Met and EMT

Chengbin Zhao^1^, Ruiming Guo^1^, Fangxia Guan^2^, Shanshan Ma^2^, Mu Li^1^, Junru Wu^1^, Xianzhi Liu^1^, Hongwei Li^1^*, Bo Yang^1^*

^1^ Department of Neurosurgery, The First Affiliated Hospital of Zhengzhou University, Zhengzhou 450052, Henan, China

^2^ School of Science, Zhengzhou University, Zhengzhou 450001, Henan, China.

Supplementary Table S1. Univariate and multivariate analyses the data from TCGA online data set (<http://cancergenome.nih.gov/>). Univariate and multivariate analyses showed that miR-128-3p was significantly associated with glioma prognosis.

| Variables | Univariate model | | | | Multivariate model | | | |  |
| --- | --- | --- | --- | --- | --- | --- | --- | --- | --- |
|  | HR | 95% CI of H | P-Value | HR | | 95% CI of H | P-Value |  | |
| miR-128-3p | 0.735 | 0.64-0.85 | 2.00E-15 | 0.846 | | 0.73-0.97 | 0.021 |  | |
| Age | 1.059 | 1.04-1.07 | 5.95E-15 | 1.058 | | 1.04-1.07 | 2.81E-13 |  | |
| Gender | 1.091 | 0.76-1.56 | 0.631 | 1.038 | | 0.73-1.49 | 0.837 |  | |
| Grade | 2.274 | 2.27-5.01 | 1.55E-09 | 2.451 | | 1.61-3.74 | 3.20E-05 |  | |
| Subtype | 1.013 | 0.71-1.45 | 1.30E-02 | 0.782 | | 0.60-1.02 | 0.065 |  | |
